# Correction to: *Opaque1* Encodes a Myosin XI Motor Protein That Is Required for Endoplasmic Reticulum Motility and Protein Body Formation in Maize Endosperm

**DOI:** 10.1093/plcell/koaf215

**Published:** 2026-01-19

This is a correction to Guifeng Wang, Fang Wang, Gang Wang, Fei Wang, Xiaowei Zhang, Mingyu Zhong, Jin Zhang, Dianbin Lin, Yuanping Tang, Zhengkai Xu, Rentao Song, *Opaque1* Encodes a Myosin XI Motor Protein That Is Required for Endoplasmic Reticulum Motility and Protein Body Formation in Maize Endosperm, *The Plant Cell*, Volume 24, Issue 8, August 2012, Pages 3447–3462, https://doi.org/10.1105/tpc.112.101360.

A reader alerted the Editors to concerns regarding Figure 6B, which concerns have also been raised via PubPeer (https://pubpeer.com/publications/761FEF9161756834AEC667857349D5). As part of the inquiry into Figure 6B, the authors confirmed that in the originally published version of this article, an error occurred in the assembly of Figure 6B. Specifically, the image of the blot was inadvertently distorted in Lane1 with mis-overlapping background during figure preparation. Upon review, the authors provided the original, unprocessed blot data, which supports the published findings and confirms that the problem arose from a digital distortion and does not alter the interpretation or conclusions of the study.

To ensure full transparency and maintain the integrity of the scientific record, we are publishing the corrected figure alongside the original blot image. The corrected version of the figure appears below.

Original Panel

**Figure:**
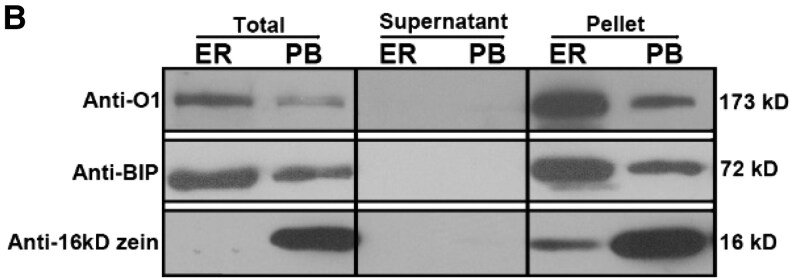


Corrected Panel

**Figure:**
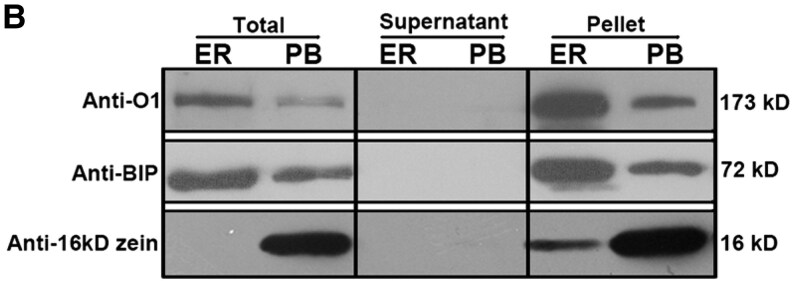


The original, unprocessed blot data of Anti-16 kD zein

**Figure:**
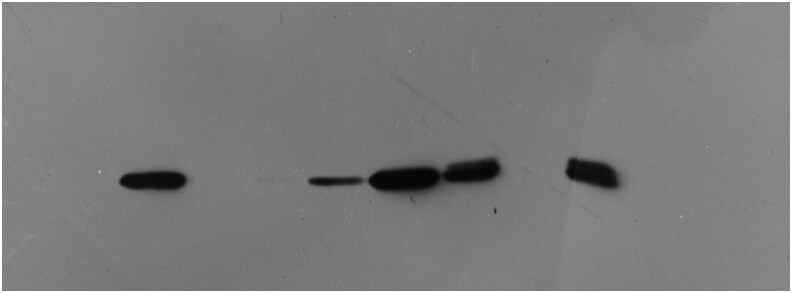


The authors apologize for this error and any confusion it may have caused. The conclusions of the article remain unchanged.

These details have been corrected only in this correction notice to preserve the published version of record.

